# Idiopathic Gingival Enlargement: A Report of a Rare Case

**DOI:** 10.7759/cureus.93121

**Published:** 2025-09-24

**Authors:** Sandip Vasave, Onkar A Bagade, Jyoti Bhavthankar, Prapti Kole

**Affiliations:** 1 Oral Pathology and Microbiology, Sir JJ Group of Hospitals, Mumbai, IND; 2 Periodontology, Yogita Dental College and Hospital, Khed, IND; 3 Oral Pathology and Microbiology, Government Dental College and Hospital, Chhatrapati Sambhajinagar, IND; 4 Oral and Maxillofacial Surgery, Swargiya Dadasaheb Kalmegh Smruti Dental College and Hospital, Nagpur, IND

**Keywords:** congenital familial fibromatosis, gingivectomy, hereditary gingival enlargement, idiopathic gingival enlargement, idiopathic gingival fibromatosis

## Abstract

Gingival fibromatosis is a heterogeneous group of disorders characterized by the progressive enlargement of the gingiva, caused by an increase in subepithelial connective tissue elements. Idiopathic gingival enlargement (IGE) is a diagnosis by exclusion when no causative agent is identified and there is no family history. This case report presents the diagnosis and treatment of a 23-year-old female patient with IGE. The patient reported a generalized diffuse gingival enlargement for the past seven years, with currently extraoral swelling on the right side and difficulty in chewing with tooth mobility. The patient was apparently healthy seven years ago when she first noticed gingival enlargement with focal areas of involvement, which gradually increased in size to the present extent, with no notable medical history and no family history. The patient reported to the private practitioner 1.5 years ago and had a localized surgical excision of the lesion, but the condition did not resolve. Radiological investigations, including an orthopantomography (OPG) and cone-beam computed tomography (CBCT), blood investigations, and an incisional biopsy of the lesion were performed. The biopsy report confirmed the diagnosis of gingival hyperplasia. Gingivectomy with multiple teeth extraction in a single surgery was performed, resulting in oroantral communication in the maxillary right posterior region. An obturator was given to cover an oroantral communication.

## Introduction

Idiopathic gingival enlargement, also referred to as gingivostomatosis, elephantiasis, idiopathic fibromatosis, hereditary gingival hyperplasia, and congenital familial fibromatosis, is a rare condition with an undetermined cause [1,2]. Attached gingiva is also affected along with marginal gingiva and interdental papilla in this condition, as opposed to phenytoin-induced gingival enlargement, where gingival overgrowth is often limited to marginal gingiva and interdental papilla. The facial and lingual surfaces of the maxilla and mandible are affected, but sometimes it may be restricted to either jaw. The gingiva appears pink, firm, and almost leathery in consistency. The gingiva shows a minutely pebbled surface. In extreme cases, the enlargement protrudes into the oral vestibule and entirely covers the teeth. As a result, the jaw appears distorted. The gingival margin may show secondary inflammatory changes [3].

On histological examination, it shows a thickened and acanthotic surface epithelium with elongated rete pegs. There is a bulbous increase in the connective tissue. It is relatively avascular with densely arranged collagen fibers and numerous fibroblasts [4].

Although many etiologies have been suggested for this condition, in most cases the mode of inheritance follows an autosomal recessive pattern, with some cases exhibiting an autosomal dominant mode of inheritance. The enlargement usually begins with the eruption of teeth, either deciduous or permanent, and may regress after extraction, suggesting that teeth or bacterial plaque associated with them may be an initiating factor or complicating factor [1-4].

## Case presentation

**Figure 1:**
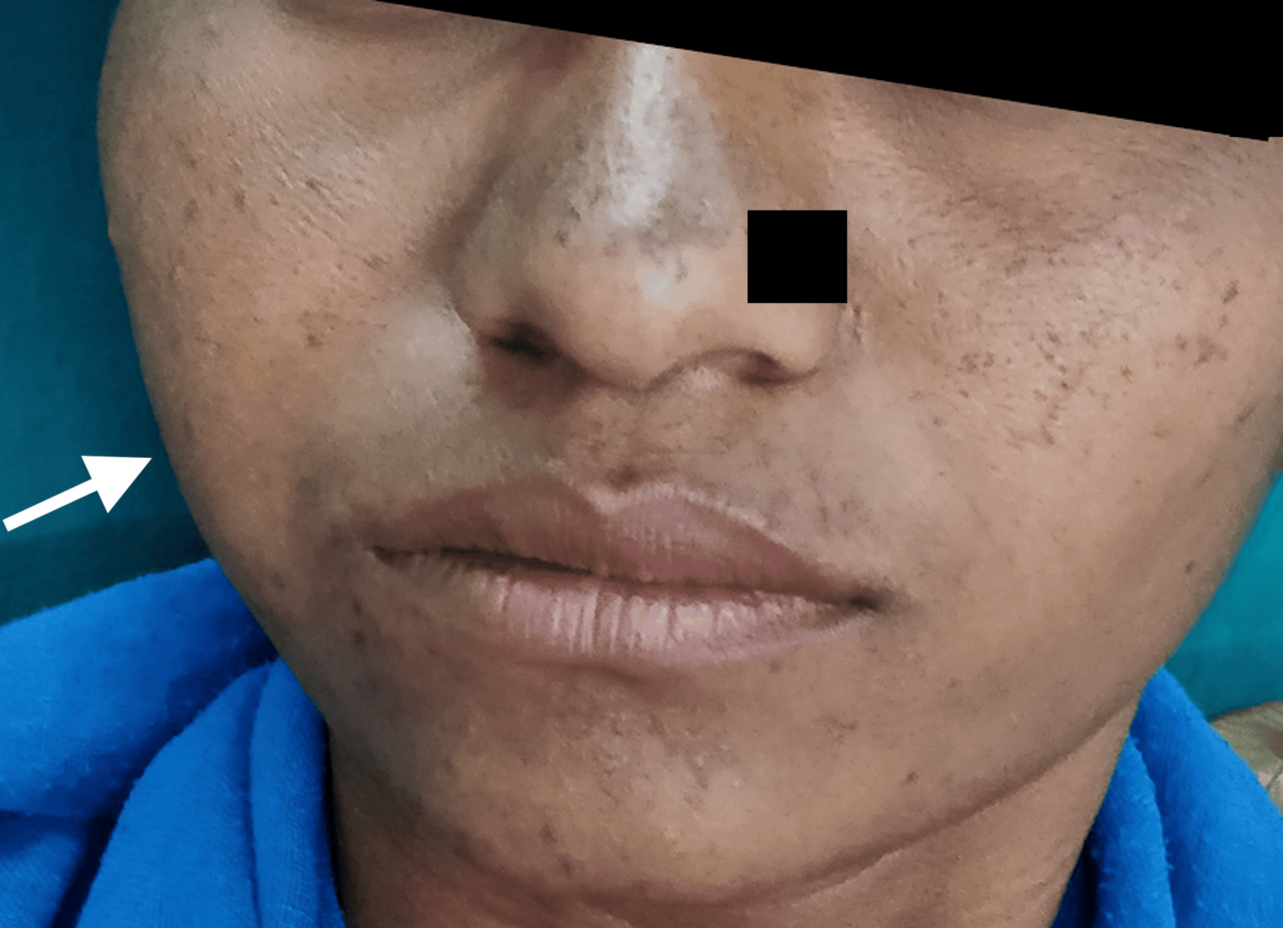
Extraoral swelling on the right side of the face

A 23-year-old female patient reported with a generalized diffuse gingival enlargement for the past seven years, with currently extraoral swelling on the right side and difficulty in chewing with multiple tooth mobility (Figure 1). The patient was apparently healthy seven years ago; then, she noticed focal areas of gingival enlargement, which gradually enlarged to their present size. The patient had undergone localized excision of the gingival enlargement at a private practice 1.5 years ago. The patient had no prior medical history of any disease or medication use.

**Figure 2:**
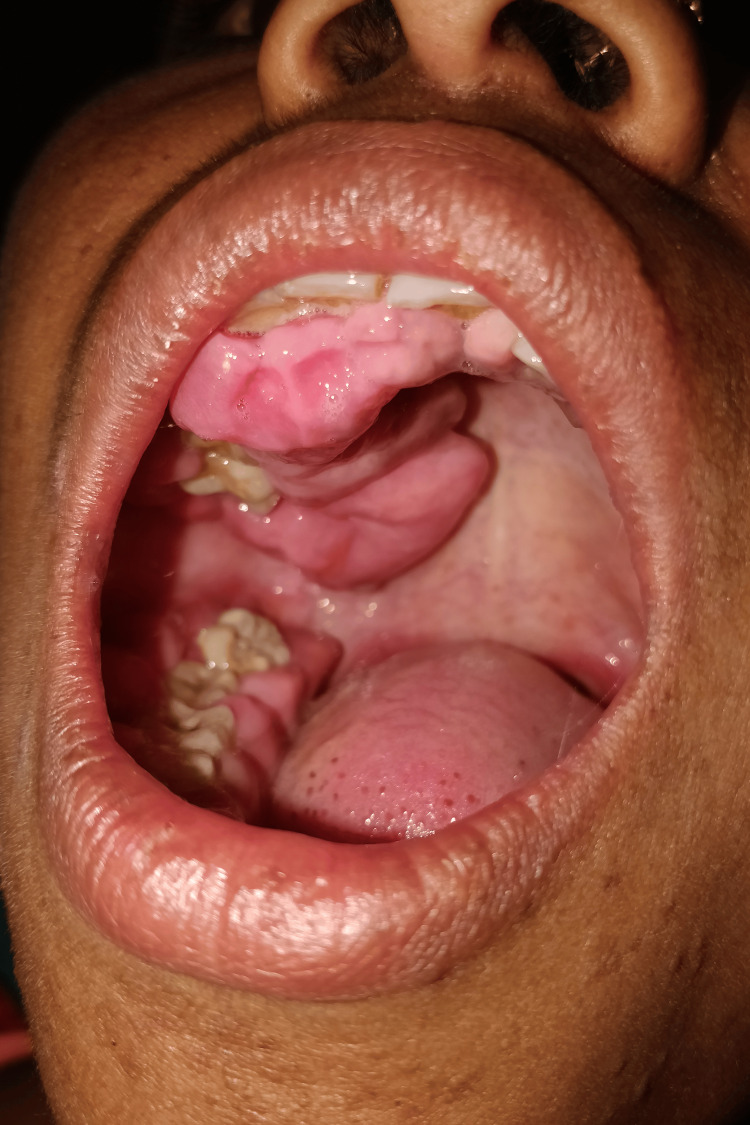
A generalized gingival swelling, prominent on the right gingiva, obliterating the complete buccal vestibule on the right side, and extending up to the mid-palatine raphe

On intraoral examination, a generalized gingival swelling was noted, prominent on the right side, obliterating the complete buccal vestibule and extending up to the mid-palatine raphe (Figure 2). The swelling was soft to firm, nontender, and friable, with a smooth surface texture and ill-defined borders. Multiple mobile teeth were present with grade III mobility in teeth 15-17 and 48; grade II mobility in teeth 11-14, 21, 22, and 24-26; and grade I mobility in teeth 23, 31, 32, 41, and 42. Tenderness on percussion was present in multiple mobile teeth. The patient's oral hygiene was poor, with the presence of plaque and calculus.

On extraoral examination, an extra-oral painless swelling was seen in the right cheek region with diffuse margins and no tenderness, with normal skin color extending from the infra-orbital margin to the zygomatic process (Figure 1). It was soft in consistency, and the overlying temperature was afebrile. The hematological parameters were within normal limits, HBsAg was negative, and the HIV antibody was nonreactive (Table 1).

**Table 1:** Hematological parameters and HBsAg rapid test results SGOT = Serum Glutamic-Oxaloacetic Transaminase, SGPT = Serum Glutamic Pyruvic Transaminase, U/L = Units per Liter, mg = milligram, HBsAg = Hepatitis B Surface Antigen

Parameter	Patient's Value (Unit)	Reference Range (Unit)
Alkaline phosphatase	97 (U/L)	36.0–113.0 (U/L)
Creatinine	0.8 (mg%)	0.6–1.4 (mg%)
Random blood sugar	107 (mg%)	70.0–140.0 (mg%)
SGOT	28 (U/L)	8.0–40.0 (U/L)
SGPT	25 (U/L)	6.0–36.0 (U/L)
Total bilirubin	0.7 (mg%)	0.1–1.2 (mg%)
Urea	28 (mg%)	15.0–45.0 (mg%)
HBsAg rapid test	Negative	Positive/Negative

**Figure 3:**
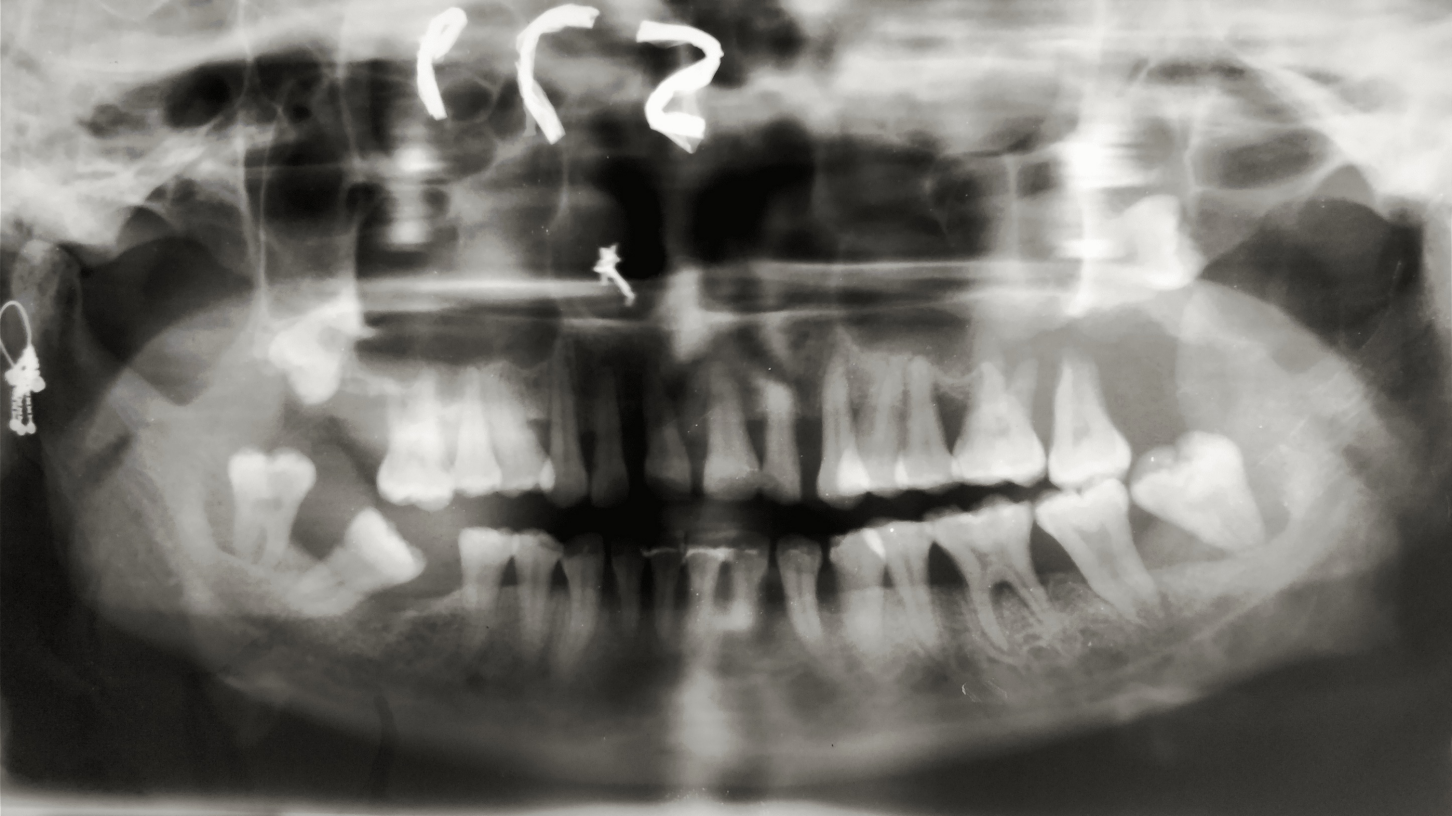
OPG showing generalized bone resorption and root resorption with tooth no. 16. The floor of the maxillary sinus was not traceable, with impacted teeth 18 and 28, with root portions extending into the maxillary sinus. A generalized interdental bone loss was observed in the mandible, with severe bone loss in teeth 37, 38, 47, and 48, as well as displaced teeth 37, 38, and 48 OPG = Orthopantomogram

**Figure 4:**
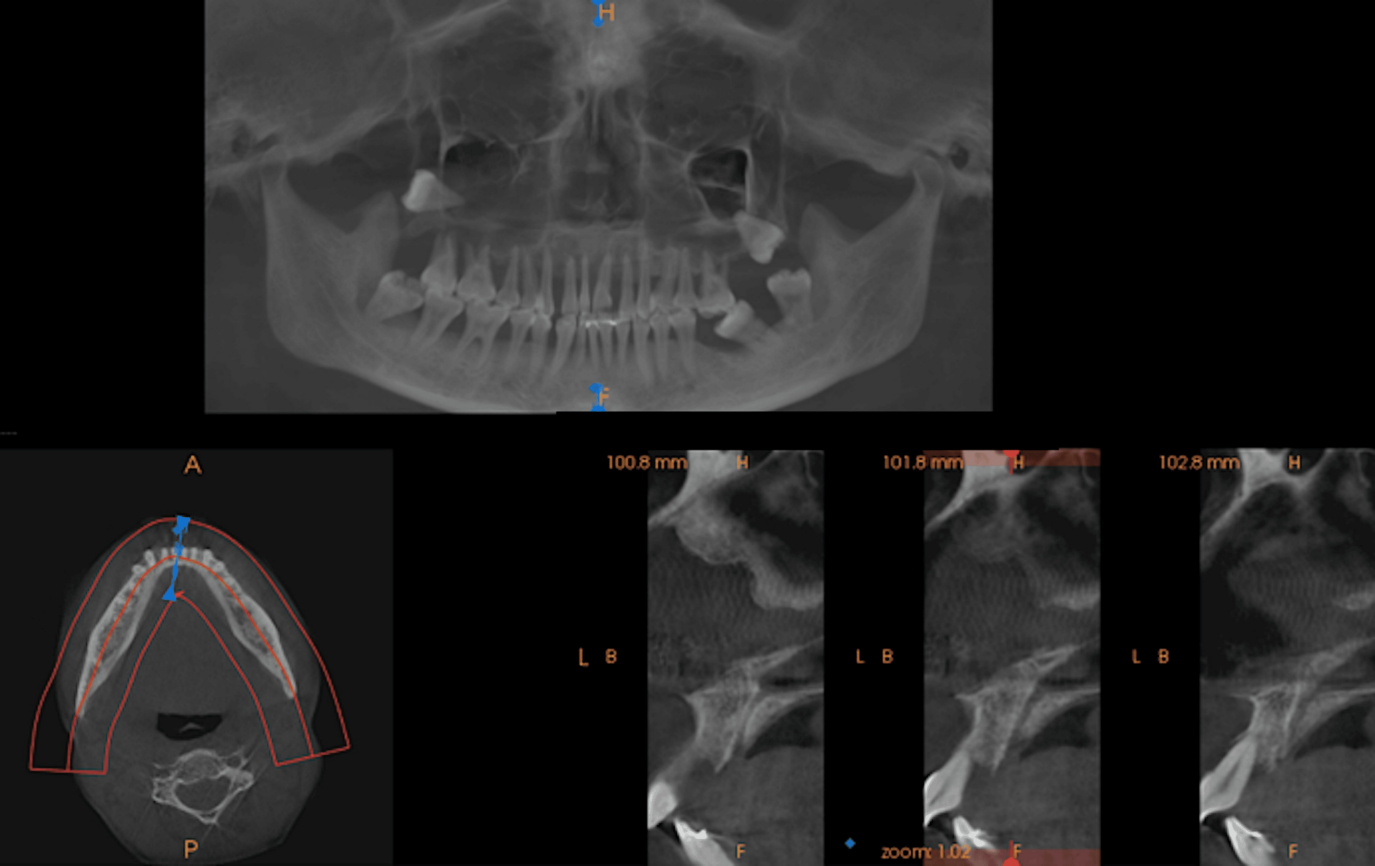
The CBCT revealed generalized bone resorption and root resorption in teeth 16 and 17. The floor of the maxillary sinus was not traceable, with impacted teeth 18 and 28, with root portions extending into the maxillary sinus. A generalized interdental bone loss was observed in the mandible, with severe bone loss in teeth 37, 38, 47, and 48, and displaced teeth 37, 38, and 48 CBCT = Cone-Beam Computed Tomography

On radiological examination, orthopantomography (OPG) and cone-beam computed tomography (CBCT) showed generalized bone resorption and root resorption with teeth 16 and 17. The floor of the maxillary sinus was not traceable, with impacted teeth 18 and 28, with root portions extending into the maxillary sinus. A generalized interdental bone loss was seen in the mandible with severe bone loss with teeth 37, 38, 47, and 48, as well as displaced teeth 37, 38, and 48 (Figures 3, 4). A provisional diagnosis of idiopathic gingival enlargement was made based on a detailed case history and radiological examination.

A small tissue specimen measuring 0.5 mm × 0.5 mm from the upper jaw was sent for biopsy. The histopathological examination showed an H&E-stained section of single soft tissue that exhibits parakeratinized stratified squamous epithelium of variable thickness; spongiosis was evident in the superficial layer of the epithelium, and the epithelium was atrophic at the underlying connective tissue.

**Figure 5:**
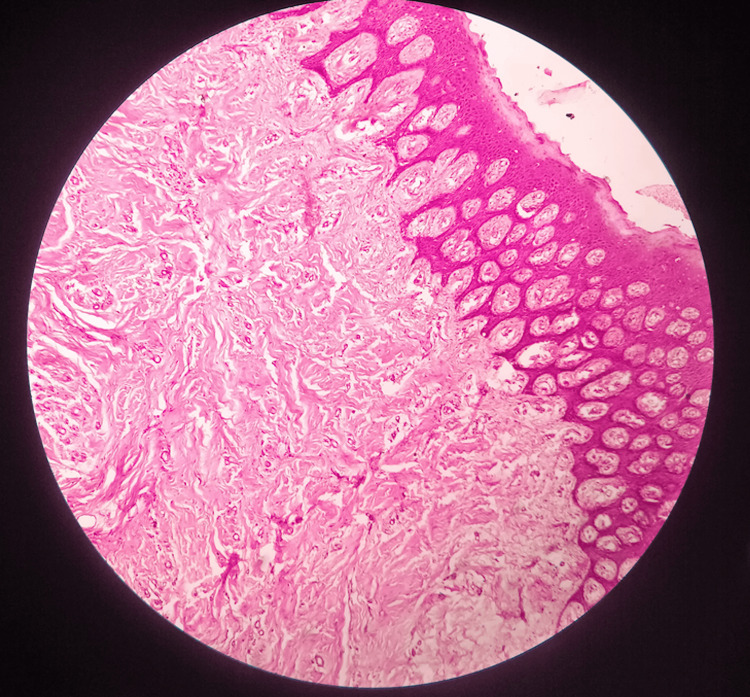
The histopathological examination showed an H&E-stained section of a single soft tissue, which exhibits parakeratinized stratified squamous epithelium of variable thickness with underlying connective tissue H&E = Hematoxylin and Eosin

The underlying connective tissue stroma showed fibrous hyperplasia, with haphazardly arranged fiber bundles. Deeper areas of the stroma exhibited a chronic inflammatory cell infiltrate consisting of plasma cells, lymphocytes, scanty eosinophils, and foam cells. A mild degree of vascularity was evident. Overall, the features were suggestive of chronic inflammatory hyperplasia (Figure 5). The diagnosis of idiopathic gingival enlargement was confirmed based on histopathology correlated with non-significant medical and family history.

**Figure 6:**
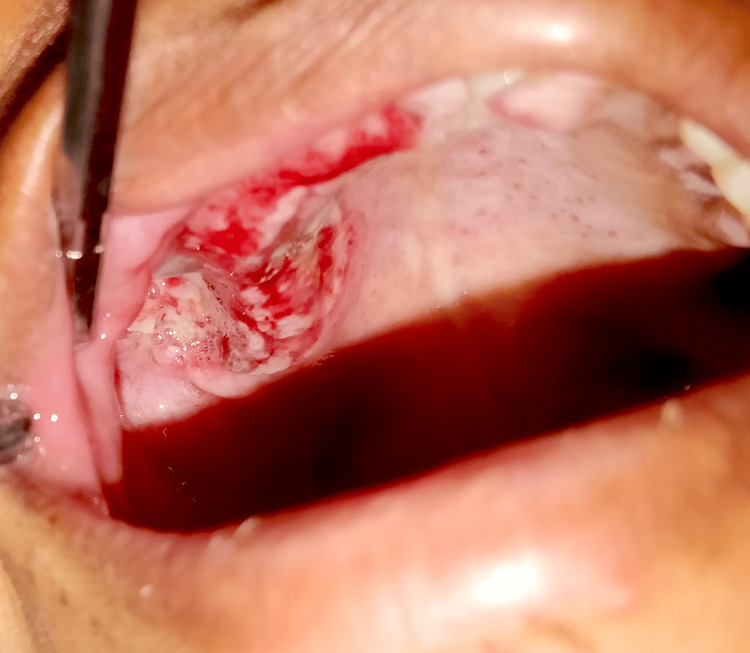
Gingivectomy with internal bevel incision

**Figure 7:**
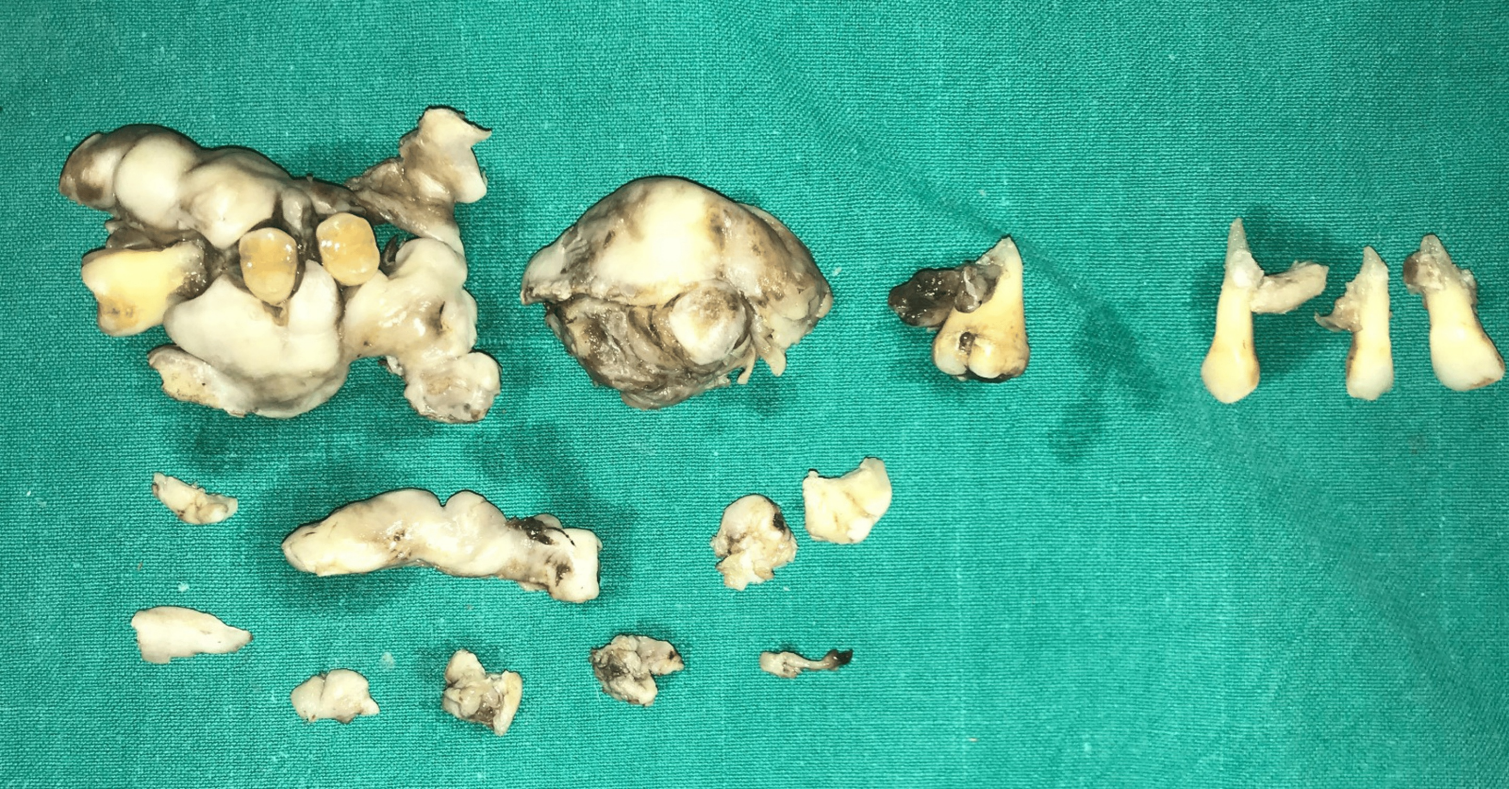
Multiple teeth extracted in a single surgery

**Figure 8:**
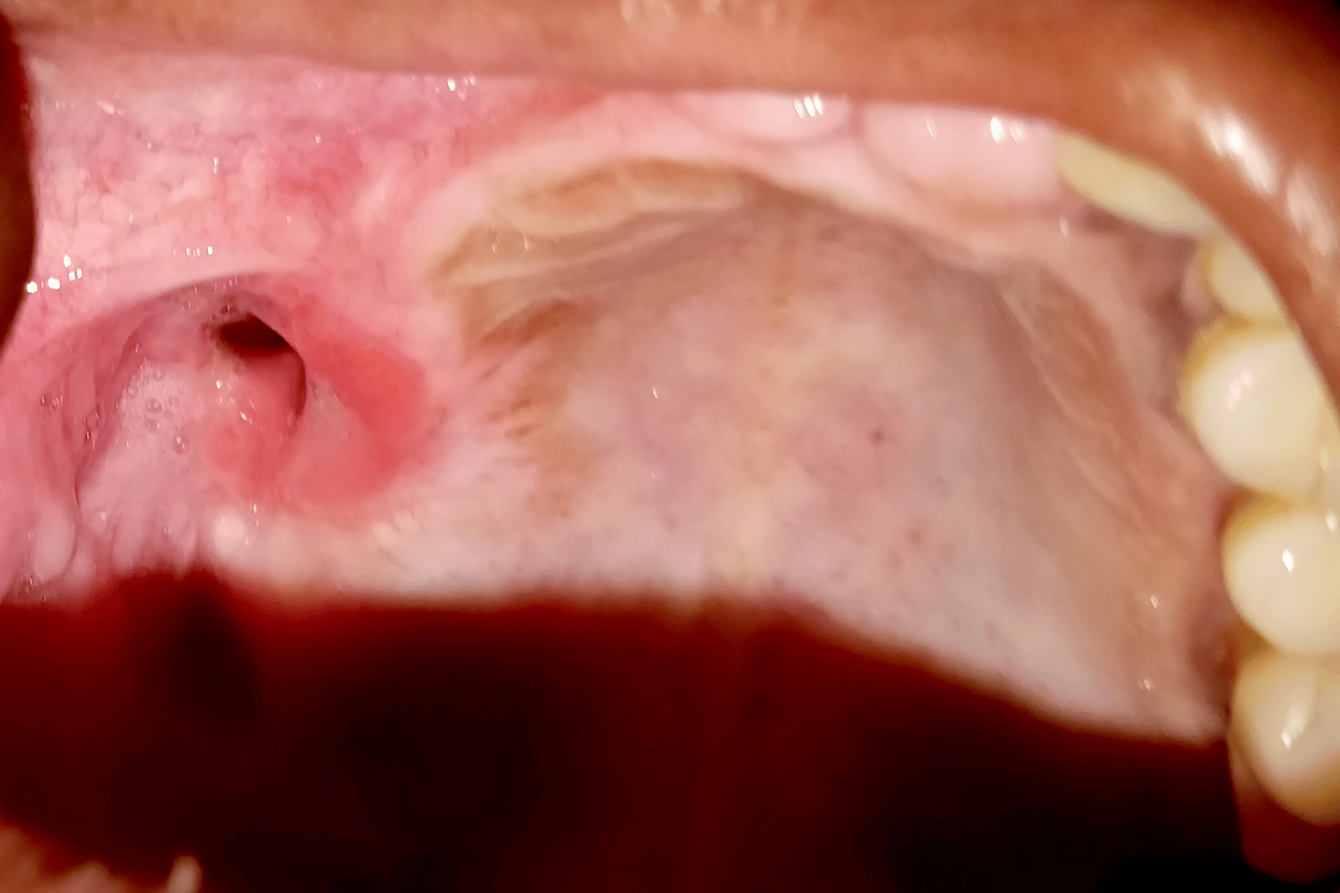
An oroantral communication formed in the maxillary posterior region

**Figure 9:**
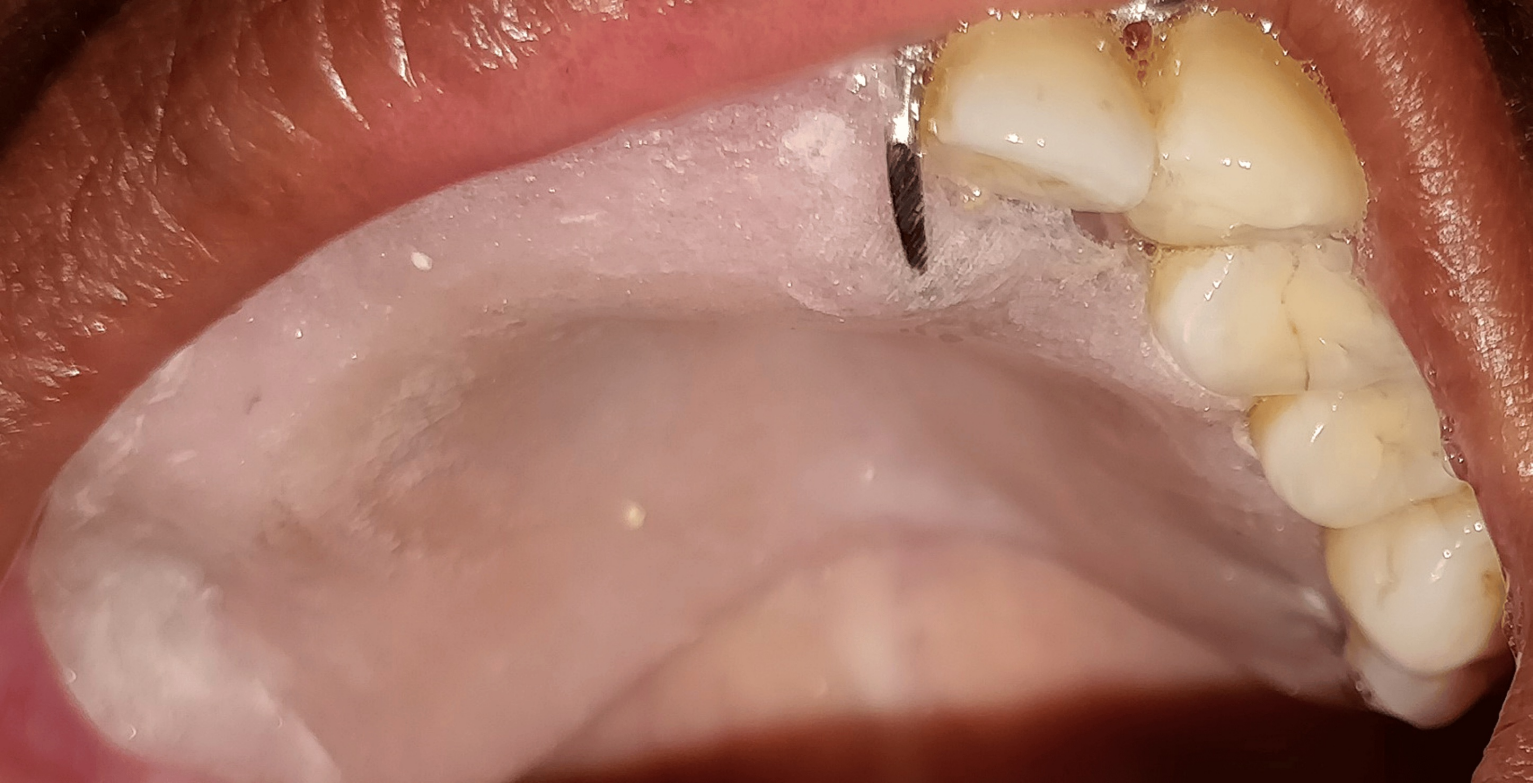
An obturator was given to cover an oroantral communication

Under local anesthesia with lignocaine and 1:80000 adrenaline, a gingivectomy with an internal bevel incision followed by multiple teeth extraction in a single surgery was performed, which led to oroantral communication in the maxillary right posterior region (Figures 6-8). An obturator was given to cover an oroantral communication (Figure 9).

## Discussion

Gingival hyperplasia presents with a varied etiology, ranging from inflammatory to drug-induced, associated with systemic conditions or diseases, to gingival fibromatosis. Various causes of gingival inflammation include trauma, chemicals, extreme temperatures, ionizing radiation, viruses, fungi, and immune defects, but currently, gingival pathologies are confined to causes by dental plaques [5,6].

Initially, based on the type of irritant, a slight ballooning of the papilla or marginal gingiva was observed, followed by an increase in size and extent to become generalized enlargement [1]. Usually, the consistency of the gingiva is soft to firm and friable at margins. It shows a smooth and shiny surface that bleeds easily [1]. Sometimes, chronic inflammatory enlargement may also appear to be firm, resilient, pink, and fibrotic enlargement, and its histology shows a high quantity of fibroblasts and collagen fibers [1].

In the present case, we did not observe plaque deposits corresponding to the gingival size. Drug-induced gingival overgrowth (DIGO) is caused by anticonvulsants, immunosuppressants, and calcium channel blockers and is usually seen within two to four months of drug usage [1]. On initial presentation, DIGO usually appears as a bead-like enlargement of the interdental papilla and may eventually involve marginal gingiva. In the absence of secondary inflammation, the bleeding on probing is absent, and the gingiva presents as mulberry-shaped, firm, pink, and resilient [1]. It resolves after tooth extraction and is absent in the edentulous areas. The DIGO due to cyclosporin shows more vascularity than due to phenytoin. The treatment includes a change of drug and, in some cases, gingivectomy or gingivoplasty [1].

In this case, there was no recorded drug history. Various syndromes are also associated with gingival hyperplasia. Gingival fibromatosis can be hereditary or idiopathic in nature. The genetic cause of hereditary gingival fibromatosis is well established, with a clear association between specific genes and different genetic loci in various populations. Various syndromes are associated with hereditary gingival fibromatosis, including prune-belly syndrome, Jones syndrome, Cross syndrome, Ramon syndrome, gingival fibromatosis with distinctive facies syndrome, Rutherfurd syndrome, Murray-Puretic-Drescher syndrome (also known as juvenile hyaline fibromatosis), and Zimmermann-Laband syndrome. In contrast, idiopathic gingival enlargement has not been associated with any specific gene or specific cause; thus, this condition is designated as idiopathic. Tuberous sclerosis, an autosomal dominant genetic disorder, has a triad of epilepsy, mental deficiency, and cutaneous angiofibromas, which also shows gingival enlargement. It exhibits an autosomal dominant inheritance pattern, resulting from a mutation in the tumor suppressor genes *TSC1* or *TSC2*, with a prevalence of approximately 1 in 6,000 individuals [7-10]. The present case did not indicate the presence of any syndrome, and a diagnosis of IGE was made by exclusion.

## Conclusions

In conclusion, this case highlights the diagnostic challenges and complexity in the management of IGE. It also underscores the importance of a multidisciplinary approach, with individualized treatment strategies aimed at addressing specific challenges in the management of IGE.

This case, involving an internal bevel gingivectomy, resulted in a favorable functional and aesthetic outcome for the patient, with the requirement for long-term follow-up to screen for recurrence.
